# Structural basis of small RNA hydrolysis by oligoribonuclease (*Cps*ORN) from *Colwellia psychrerythraea* strain 34H

**DOI:** 10.1038/s41598-019-39641-0

**Published:** 2019-02-25

**Authors:** Chang Woo Lee, Sun-Ha Park, Chang-Sook Jeong, Sun-Shin Cha, Hyun Park, Jun Hyuck Lee

**Affiliations:** 1 Unit of Polar Genomics, Korea Polar Research Institute, Incheon, 21990 Republic of Korea; 2 Department of Polar Sciences, University of Science and Technology, Incheon, 21990 Republic of Korea; 3 Department of Chemistry & Nanoscience, Ewha Woman’s University, Seoul, 03760 Republic of Korea

## Abstract

Cells regulate their intracellular mRNA levels by using specific ribonucleases. Oligoribonuclease (ORN) is a 3′–5′ exoribonuclease for small RNA molecules, important in RNA degradation and re-utilisation. However, there is no structural information on the ligand-binding form of ORNs. In this study, the crystal structures of oligoribonuclease from *Colwellia psychrerythraea* strain 34H (*Cps*ORN) were determined in four different forms: unliganded-structure, thymidine 5′-monophosphate *p*-nitrophenyl ester (*p*NP-TMP)-bound, two separated uridine-bound, and two linked uridine (U-U)-bound forms. The crystal structures show that *Cps*ORN is a tight dimer, with two separated active sites and one divalent metal cation ion in each active site. These structures represent several snapshots of the enzymatic reaction process, which allowed us to suggest a possible one-metal-dependent reaction mechanism for *Cps*ORN. Moreover, the biochemical data support our suggested mechanism and identified the key residues responsible for enzymatic catalysis of *Cps*ORN.

## Introduction

The accuracy of RNA processing, quality control, and turnover are critical in all organisms. Among the various stages of RNA metabolism, RNA degradation is a crucial step in both prokaryotic and eukaryotic cells. In response to environmental signals, cells regulate the intracellular levels of mRNA, using specific ribonucleases^1^. This plays a key role in regulating gene expression and protein synthesis. In bacteria, the lifetime of mRNAs is much shorter than that in eukaryotes. The limited lifetime of mRNA in bacteria enables the cells to regulate protein synthesis continuously and rapidly in response to its varying requirements^2–5^.

To date, identification and characterisation of ribonucleases, enzymes that hydrolyse the phosphodiester bond in nucleic acids, have been carried out primarily using *Escherichia coli* as a model organism. Only recently, the investigation of ribonucleases has expanded to other prokaryotes and eukaryotes. In *E. coli*, degradation of mRNA is initiated by endoribonucleases that cleave the bonds of RNA from within the molecule. A major endoribonuclease involved in this process is RNase E, which is a component of a multiprotein complex, the RNA degradosome^2,3,6–8^. Endoribonucleases RNase G and RNase III also participate in the initiation of mRNA degradation^9^. The cleaved mRNA fragments are further cleaved by 3′–5′ exoribonucleases. RNase II, RNase R, and PNPase are examples of 3′–5′ exoribonucleases that cleave the nucleotides from the 3′-end of the RNA^10,11^. However, these exoribonucleases are incapable of complete mRNA degradation, as they are inactive against short oligonucleotides (pN)_n_ (n ≤ 5 bases). Therefore, oligoribonucleases (ORN), RNases targeted toward short oligonucleotides, are needed for the complete digestion of short oligonucleotides into mononucleotides^12^. A defective ORN leads to various problems including the accumulation of short mRNA fragments in the cell^13^. Among the eight known exoribonuclease genes in *E. coli*, *orn* gene is the only one essential for survival^14^. Recent studies have shown that *orn* deletion mutant of *Pseudomonas aeruginosa* cannot degrade 5′-phosphoguanylyl-(3′, 5′)-guanosine (pGpG), resulting in the accumulation of cyclic diguanylate (c-di-GMP). In addition, the *orn* deletion mutant of *P. aeruginosa* shows diminished cytotoxicity, by virtue of a malfunctioning in the type III secretion system^15–17^.

Exoribonucleases are classified into six superfamilies (RNR, DEDD, RBN, PDX, RRP4, and 5PX) based on common structural features derived from sequence analysis^18^. Among these, ORN belongs to the DEDD superfamily, comprising 3′-5′ exonucleases that require a divalent cation for activity^19–21^. The DEDD superfamily proteins contain specific motifs (ExoI, ExoII, and ExoIII) and four specific, invariant acidic residues. In addition to ORNs, the proofreading domains of DNA polymerases and DNA exonucleases are also members of this superfamily^22–25^.

Several crystal structures of ORN have been reported, such as those of *Xanthomonas campestris* ORN (PDB code 2GBZ), *Coxiella burnetii* ORN (PDB code 3TR8), *Haemophilus influenzae* ORN (PDB code 1J9A; literature describing the structure is not yet published) and *E. coli* ORN (PDB code 1YTA; literature describing the structure is not yet published)^26,27^. However, there is no structural information on the ligand-binding form of ORNs to date. Therefore, the structural conformation and functional requirements for RNA-binding remain unclear.

Our research group have focused on the mechanism of action of oligoribonuclease (*Cps*ORN) from the psychrophilic gram-negative bacterium *Colwellia psychrerythraea* strain 34H, to understand how the enzyme hydrolyses small nucleic acids^28^. Cold-active enzymes have high catalytic efficiency at low and moderate temperatures and are suitable for biotechnological applications. We have identified several beneficial cold-active enzymes from *C. psychrerythraea*, which is an obligate psychrophile isolated from the Arctic marine sediments whose genome has been completely sequenced, in the last ten years^29–34^. Cold-active ORN could be used to completely digest small RNA molecules in molecular biology and cell biology experiments. Therefore, *Cps*ORN was chosen as the target protein for biochemical and structural studies.

In this study, we cloned, purified, and characterised the enzyme activity of *Cps*ORN (UniProtKB code Q47VZ4). Furthermore, crystal structures of unliganded *Cps*ORN (wild-type), thymidine 5′-monophosphate *p*-nitrophenyl ester (*p*NP-TMP)-bound (D163A inactive mutant) form, two separated uridine-bound form (wild-type), and two linked uridine (U-U)-bound form (D163A inactive mutant) were determined to obtain the structural information and to examine its enzymatic mechanism at the molecular level. Structural analysis results show that *Cps*ORN is a stable dimer and its active site is formed by swapping residues from the other subunit. Structural comparisons of the apo-form and the RNA-bound form revealed several conformational changes induced by ligand-binding. In addition, several active site residues of *Cps*ORN were mutated and the effects on RNA-binding and hydrolysis were assessed to test the structural predictions and identify residues that are important for enzymatic action. This structural information together with the biochemical assay data provides novel insights into the substrate-binding mode and one-metal-dependent exo-ribonuclease mechanism of *Cps*ORN.

## Results and Discussion

### Oligoribonuclease properties of *Cps*ORN

**Figure 1:**
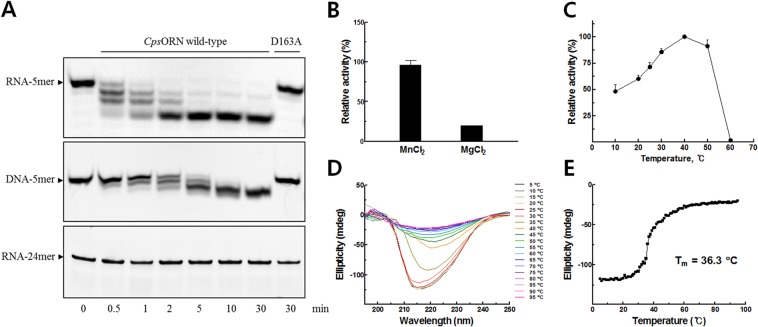
Characterisation and oligoribonuclease activity of *Cps*ORN. (**A**) Time course of oligoribonuclease activity of *Cps*ORN wild-type and D163A mutant on 5′- fluorescein-labelled RNA or DNA substrates. The reaction mixture contained 50 mM Tris (pH 8.0), 5 mM MnCl_2_, 20 ng protein, and 100 pmol substrate. The substrates are as follows: top panel, 5′-F-UUUUU-3′, middle panel, 5′-F-TTTTT-3′, and bottom panel, 5′-F-CACACACACACACACACACACACA-3′ (F denotes a fluorescein). Full-length gels are presented in Supplementary Fig. S2 (**B**) Comparison of the metal-dependent activities of *Cps*ORN. The hydrolysis activity of *p*NP-TMP was determined in the presence of MnCl_2_ or MgCl_2_ (1 mM). (**C**) Effects of temperature on the hydrolysis of *p*NP-TMP by *Cps*ORN. The enzymatic reactions were performed at various temperatures under standard conditions. The relative activities are expressed as percentages of the maximum activity and the error bars represent the standard deviations of triplicate measurements. (**D**) Thermal stability of *Cps*ORN. Far-UV CD spectra of *Cps*ORN were obtained at different temperatures as indicated in Materials and Methods. *Cps*ORN in Tris-NaCl buffer was unfolded at an increasing temperature. (**E**) Melting curve monitored by CD at 222 nm to obtain the melting temperature (T_m_).

As a first step toward elucidating the exact enzymatic mechanism of *Cps*ORN, we investigated the oligoribonuclease activity of purified recombinant *Cps*ORN. Previously characterised ORNs degrade short RNA (≤5) in the 3′–5′ direction and require a divalent cation for activity^21,35,36^. Therefore, we assessed the *Cps*ORN activity using 5′-fluorescein-labelled RNA substrates to detect intermediate products of degradation in the presence of manganese ions. As shown in Fig. 1A, *Cps*ORN was able to cleave 5-mer RNA (5′-F-UUUUU-3′) to near completion after 5 min, in the 3′–5′ direction. In addition, we tested *Cps*ORN activity on oligo DNA because the enzymes belonging to the same 3′–5′ exonuclease superfamily as ORN contain the proofreading domains of DNA polymerases. As expected, we observed time course degradation of 5-mer DNA (5′-F-TTTTT-3′) but *Cps*ORN was more active on RNA than on DNA, which is consistent with a previous report^37^. Our data suggest that *Cps*ORN might participate in DNA repair as well. Furthermore, to check the possibility that *Cps*ORN could also degrade longer substrates, the activity of *Cps*ORN on 24-mer RNA (5′-F-CACACACACACACACACACACACA-3′) was tested. The degradation of 24-mer RNA was not observed, which clearly demonstrates that *Cps*ORN has a length specificity for RNA degradation (Fig. 1A).

The RNase activity of *Cps*ORN was also investigated, via the hydrolysis of *p*NP-TMP to *p*-nitrophenol and TMP^38^. *p*NP-TMP is a phosphodiester analogue of a natural nucleic acid substrate. Since pNP-TMP is a dinucleotide mimic that can be cleaved by *Cps*ORN, it is thought that *Cps*ORN may be able to degrade the 2-mer of natural RNA substrate into final 1mer molecules. *Cps*ORN showed hydrolysis activity toward *p*NP-TMP in the presence of manganese or magnesium. However, the hydrolysis activity in the presence of magnesium was less than 20% of the activity with manganese (Fig. 1B). Temperature-dependent activity change of *Cps*ORN was studied using *p*NP-TMP substrate at different temperatures in the range of 10–60 °C (Fig. 1C). The *Cps*ORN exhibited maximum activity at 40 °C and the obtained value was set to 100%. Approximately 50% relative activity was exhibited even at 10 °C, and the activity dropped sharply at temperatures over 50 °C. Furthermore, to examine the thermal stability of *Cps*ORN, circular-dichroism (CD) experiments were performed (Fig. 1D). *Cps*ORN is sensitive to heat treatment; at ~50 °C, the enzyme got denatured almost completely and the melting temperature was 36.3 °C (Fig. 1E). In contrast, *Eco*ORN is more stable than *Cps*ORN. *Eco*ORN shows a remarkable thermal stability even at 65 °C^12^. These results suggest that *Cps*ORN has typical properties of cold-active enzymes^39,40^.

### Overall structure of *Cps*ORN

**Figure 2:**
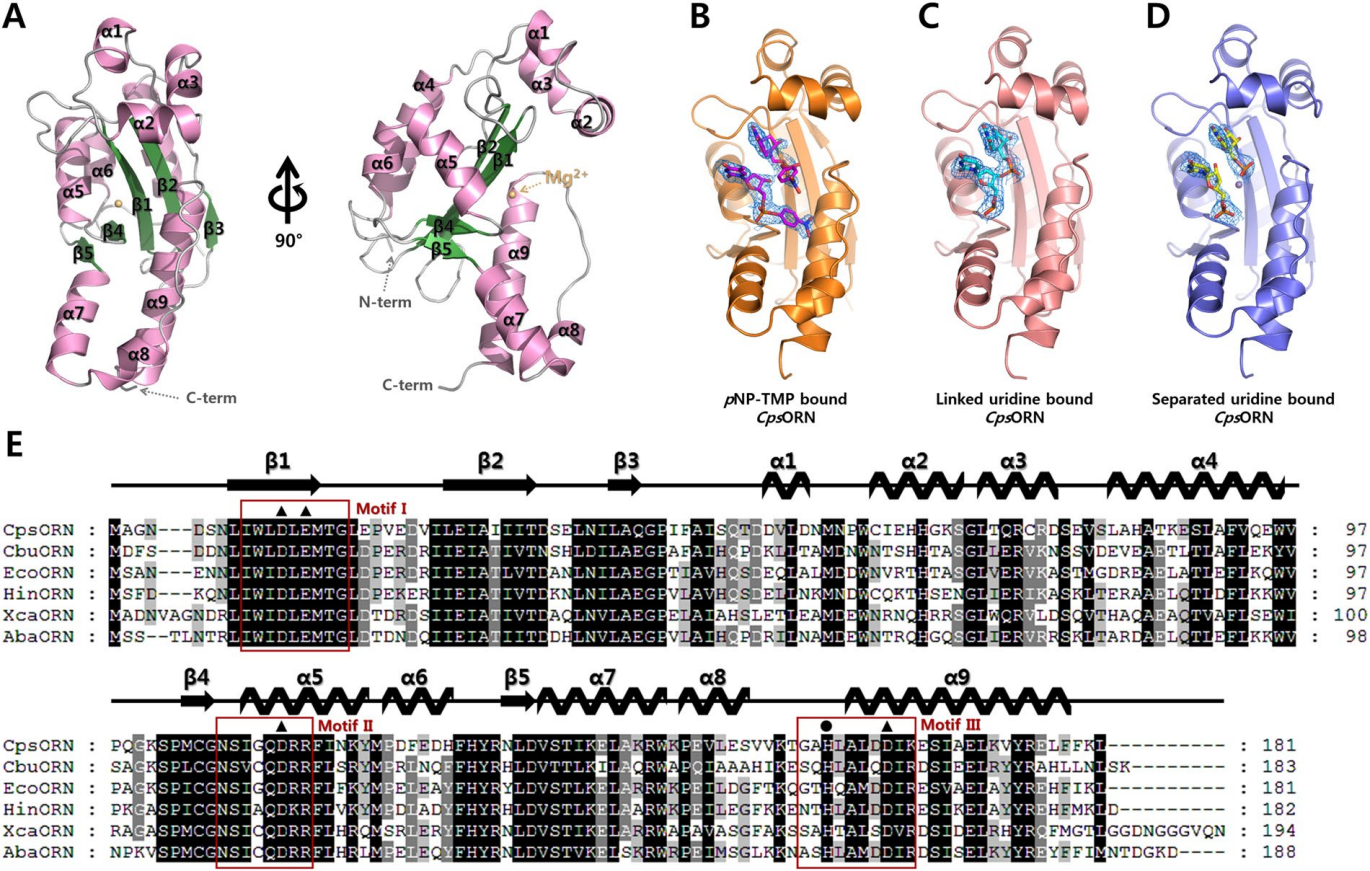
Crystal structure and multiple sequence alignment of *Cps*ORN. (**A**) The overall structure of the unliganded *Cps*ORN monomer with one magnesium ion (light orange sphere) in the active site. (**B**) Ribbon diagram of the *p*NP-TMP-bound D163A inactive mutant *Cps*ORN structure. The bound ligand is shown as a stick model (magenta) with a 2*F*o-*F*c electron density map (contoured at 1σ). (**C**) Ribbon diagram of the two linked uridine (U-U)-bound *Cps*ORN structure. The bound ligand is shown as a stick model (cyan) with a 2*F*o-*F*c electron density map (contoured at 1σ). (**D**) Ribbon diagram of the two separated uridine-bound *Cps*ORN structure. The bound ligand is shown as a stick model (yellow) with a 2Fo-Fc electron density map (contoured at 1σ) (**E**) Structure-based multiple sequence alignment of *Cps*ORN with other ORNs. The secondary structures marked above the sequences are according to the unliganded CpsORN. The aligned sequences include *Cps*ORN (UniProtKB code Q47VZ4), *Cbu*ORN (UniProtKB code Q83C93, PDB code 3TR8), *Eco*ORN (UniProtKB code P0A784, PDB code 1YTA), *Hin*ORN (UniProtKB code P45340, PDB code 1J9A), *Xca*ORN (UniProtKB code Q8P8S1, PDB code 2GBZ), and *Aba*ORN (UniProtKB code V5VGJ9, PDB code 5CY4). The conserved motifs (ExoI, ExoII, and ExoIII) of the DEDD superfamily are boxed in red. The four conserved invariant acidic residues (Asp12, Glu14, Asp112, and Asp163) are indicated by filled triangles. The residue (His158) suspected as an activator of water molecule in the enzymatic reaction is indicated by the filled circle.

The crystal structures of oligoribonuclease from *C. psychrerythraea* 34H (*Cps*ORN) were determined in the unliganded form (wild-type; 2.7 Å resolution), thymidine 5ʹ-monophosphate *p*-nitrophenyl ester (*p*NP-TMP)-bound form (D163A inactive mutant; 2.2 Å resolution), two linked uridine (U-U)-bound form (D163A inactive mutant; 2.45 Å resolution), and two separated uridine-bound form (wild-type; 2.2 Å resolution) (Fig. 2A–D). First, the unliganded *Cps*ORN structure was solved by molecular replacement method, using oligoribonuclease from *Haemophilus influenzae* (PDB code 1J9A) as a template. Next, other complex forms of *Cps*ORN were solved by molecular replacement method, using the unliganded *Cps*ORN structure as the template model. The crystal structure of unliganded *Cps*ORN contains five antiparallel β-strands and eight α-helices, with a compact α/β fold (Fig. 2A). The overall structure of *Cps*ORN was similar to other reported exoribonucleases of the DEDD superfamily. Structure alignment search using DALI server showed that CpsORN exhibited high structural similarity with ORN from *E. coli* (*Eco*ORN; PDB code 1YTA), *H. influenzae* (*Hin*ORN; PDB code 1J9A), and *Acinetobacter baumannii* (*Aba*ORN; PDB code 5CY4), and XC847 from *Xanthomonas campestris* (*Xca*ORN; PDB code 2GBZ)^27^. The crystal structures of RNase T, belonging to the DEDD superfamily, also have a high structural similarity (Supplementary Table S2)^41^. *Cps*ORN has a characteristic core sequence, comprising four invariant acidic amino acids (Asp12, Glu14, Asp112, and Asp163). In addition, *Cps*ORN contains conserved motifs I, II, and III, representative of the DEDD superfamily (Fig. 2E).

**Figure 3:**
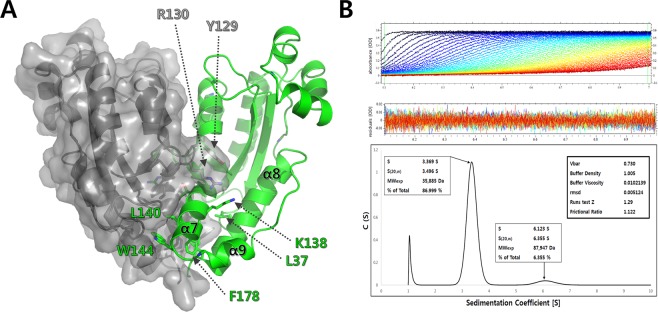
Dimerisation of *Cps*ORN (**A**) The dimerisation of *Cps*ORN is mediated by hydrophobic interactions between the α7, α8, and α9 helices. Subunit A is presented in the surface area model (grey) and subunit B is presented in the ribbon diagram (green). (**B**) Analytical ultracentrifugation (AUC) experiments indicate that *Cps*ORN is a stable dimer in solution. AUC experiments were performed using an XL-A analytical ultracentrifuge (Beckman Coulter, Brea, CA, USA) with 0.5 mg mL^−1^ of *Cps*ORN protein (residues 1–181; calculated molecular weight: 20 kDa for the polypeptide chain). *Cps*ORN protein was diluted in a buffer comprising 20 mM Tris-HCl (pH 8.0) and 150 mM NaCl. Protein solutions and the buffer were loaded into the sample and reference sectors of the dual-sector epon centrepiece, respectively. Centrifugation was performed at 45,000 rpm, and sedimentation profile was monitored at 280 nm. Sedimentation-velocity data were analysed using the SEDFIT program^52,53^. The major peak of 3.496 S was detected with a sedimentation coefficient S20 (dimer). The estimated molecular weight is 36 kDa based on AUC results, whereas the calculated molecular weight of the monomer *Cps*ORN is 20.6 kDa.

In all four *Cps*ORN crystal forms, a C2-symmetrical triangle-shaped homodimer was observed, corresponding to the physiological dimer existing in solution as shown by analytical ultracentrifugation (Fig. 3). Oligomeric states of other ORNs and RNase T have also been known to exist as dimers. *Cps*ORN forms a dimer mediated by the interaction of α7, α9 helices, and β5 strand. The α7, α8, and α9 helices protrude out of the catalytic site. These protruding α-helices are stabilised via dimer formation through hydrophobic interactions. Residues Leu37, Tyr129, Leu132, Ile137, Leu140, Trp144, Leu171, Phe178, and Phe179 participate in this hydrophobic interaction. Notably, Tyr129, Leu140, and Trp144 residues are found deeply embedded in the hydrophobic core of the other monomer. Several hydrogen bonds also contribute to the interface of dimerisation. Residues Arg130, Thr136, and Glu139 participate in the dimerisation of *Cps*ORN by forming hydrogen bonds.

### Active site of *Cps*ORN

**Figure 4:**
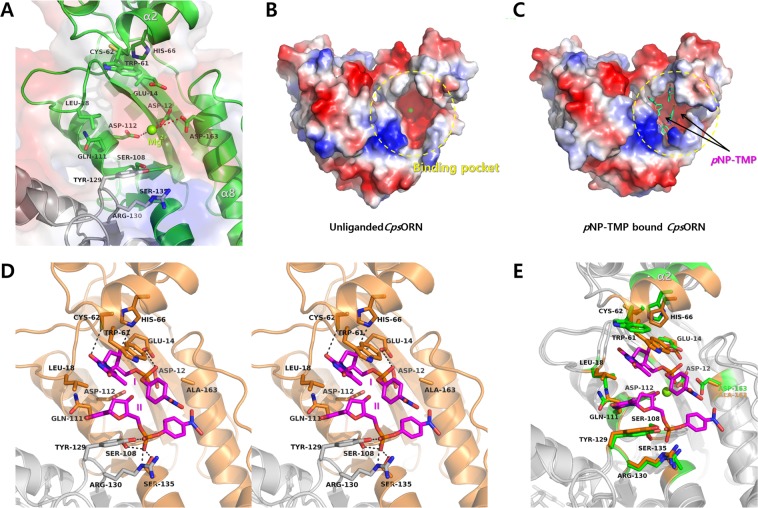
Binding of *p*NP-TMP to D163A inactive *Cps*ORN. (**A**) The active site of unliganded *Cps*ORN contains magnesium ion. The residues located in the active site are presented as a green stick model and bound magnesium ion is presented as a light green sphere. (**B**) The active site of *Cps*ORN has a negatively charged surface, created by Asp12, Glu14, Asp112, and Asp163. (**C**) Two molecules of *p*NP-TMP (lime green) bind to the active site of *Cps*ORN. Notably, D163A inactive *Cps*ORN mutant does not contain a divalent ion, which means that metal ion is not essential for ligand-binding. (**D**) Stereo view of the atomic interactions between bound *p*NP-TMP and *Cps*ORN. (**E**) Conformation changes in the *p*NP-TMP binding residues are shown by structural superposition of unliganded (green) and *p*NP-TMP-bound *Cps*ORN (orange) structures.

In the structure of unliganded *Cps*ORN, the α2, α7, α8, and α9 helices, along with the β1 and β2 strands form a negatively charged pocket. A magnesium ion, located in the centre of the pocket, tightly interacts with the invariant acidic amino acids of Asp12, Glu14, Asp112, and Asp163 residues (Fig. 4A,B). The dimer has two separated RNA-binding pockets at the opposite ends as shown Fig. 4B,C. Thus, it is thought that the two dimeric and symmetric active sites of *Cps*ORN may have similar affinity for the same substrate as they are almost identical and separated. The putative active site of *Cps*ORN was confirmed by solving the structure of the *p*NP-TMP-bound *Cps*ORN (D163A inactive mutant). The *p*NP-TMP complexed D163A mutant *Cps*ORN crystal contains two *p*NP-TMP molecules in the active site of each monomer, but no metal ion was found at the expected position of the magnesium ion (Fig. 4C,D). This is consistent with the fact that the D163A mutant has no RNase activity, and bound *p*NP-TMP is not cleaved in *p*NP-TMP complexed *Cps*ORN structure. These results also suggest that the Asp163 residue is important for metal ion coordination as well as enzyme activity. Structural alignment between the unliganded form and the *p*NP-TMP-bound D163A mutant structure of *Cps*ORN has 0.503 Å overall r.m.s deviation for 159 C_α_ atoms (Fig. 4E). The major structural differences were found in the α2 helix and the α8-α9 loop region near the active site, suggesting that ligand-binding induces these conformational changes. Detailed examination revealed that the side chain of Trp61, located on the α2 helix is flipped, and extends to the active site. This change leads to the shift of the α2 helix toward the active site. The α8-α9 loop region also shifts slightly toward the active site and interacts with the bound *p*NP-TMP. These conformational changes generate the closed state of *p*NP-TMP*-*bound *Cps*ORN compared to the unliganded form of *Cps*ORN. Each of the two bound *p*NP-TMPs (I and II) were spilt by Leu18 residue located on the β1-β2 loop region. The 3′-OH atom of the sugar moiety of *p*NP-TMP (I) interacts with NE2 of His66 and OE1 of Glu14. The side chain of Cys62 interacts with 2′-oxygen atom of the thymine base of *p*NP-TMP (I). In addition, the phosphate part of *p*NP-TMP (II) interacts with the side chain of Ser108 and Ser135. Moreover, Tyr129 and Arg130 residues from the other subunit participate in the binding of *p*NP-TMP (II) (Fig. 4D). To investigate the RNA-binding mode of *Cps*ORN, the oligoribonucleotide bound form (D163A inactive mutant) and separated RNA-bound form (wild-type) were determined. In these two different forms, the bound molecules occupy similar positions of *p*NP-TMP in the *Cps*ORN structure. Details of the interactions and binding modes are described below.

### RNA-binding of *Cps*ORN

**Figure 5:**
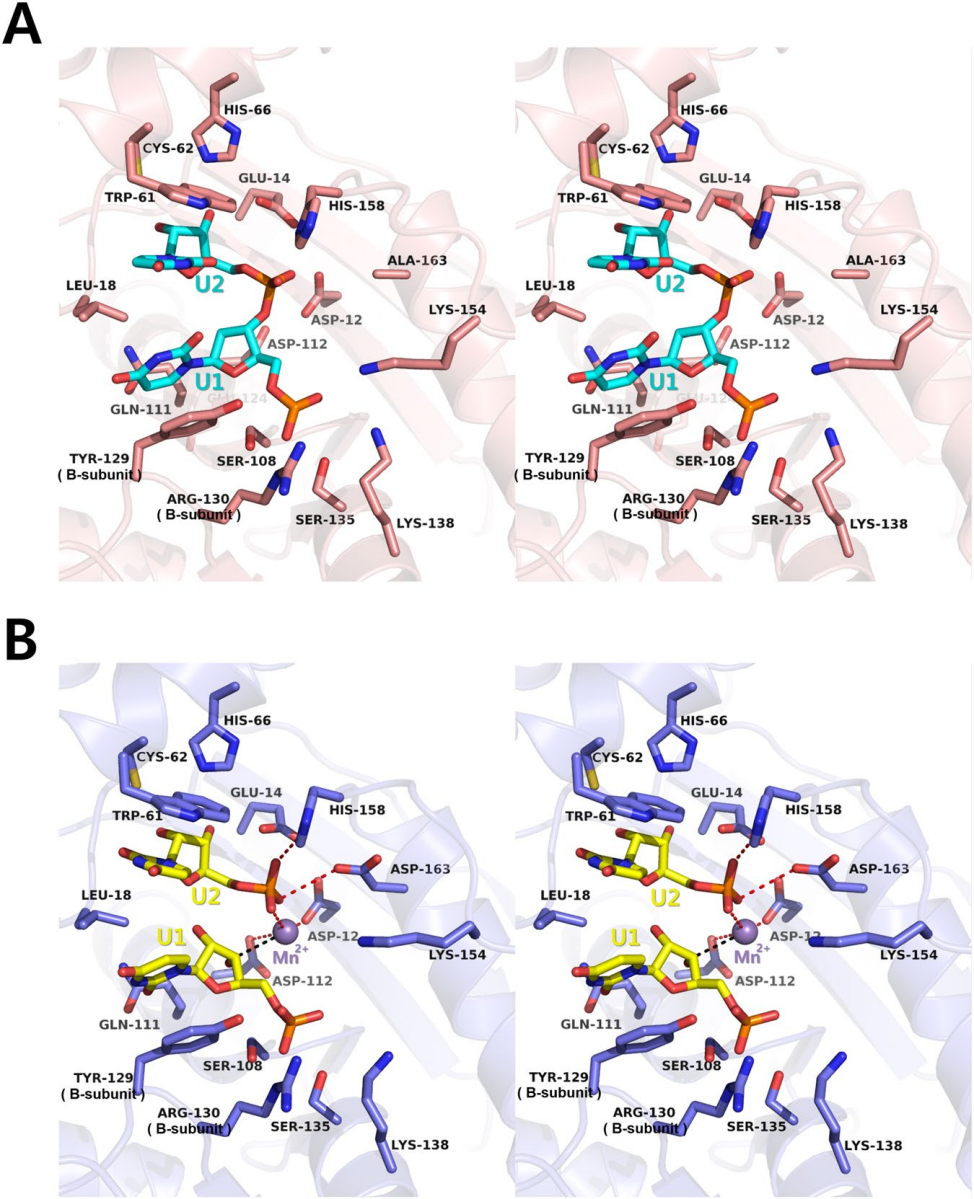
RNA-binding mode of *Cps*ORN. (**A**) Stereo view of the active site residues (salmon) and bound linked uridine (cyan). (**B**) Stereo view of the active site residues (slate blue) and separated uridine (yellow). Bound manganese ion is shown as a purple sphere.

A U-U dinucleotide and two uridine mononucleotides were clearly identified in the RNA substrate-binding site of *Cps*ORN (Fig. 5). Surface charge distribution shows that the substrate-binding site is highly negatively charged. This is essential for the polar interaction with the divalent metal ion. In the two linked uridine (U-U)-bound structure, sufficient electron density was observed for only two uridine molecules, although five linked uridine (5′-U(-3)-U(-2)-U(-1)-U(1)-U(2)-3′) was used for crystallisation. Even though a partial electron density of the third uridine was observed, the intensity of electron density was significantly weak. This means that *Cps*ORN tightly grabs only the last two nucleotides of the 5-mer RNA. Meanwhile, the rest of the 5-mer RNA are located outside the binding pocket, in a flexible state. The two linked ribonucleotides that are bound are numbered as 5′-U1-U2-3′ for further description. For this RNA-bound structure determination, D163A inactive mutant *Cps*ORN protein was used, as mentioned in the *p*NP-TMP-bound structure. The last two ribonucleotides make hydrophobic stacking interactions with Leu18, Trp61, and Tyr129 residues. A more detailed examination revealed that the indole ring of Trp61 forms a hydrophobic interaction with the base of nucleotide U2, forming a barrier at the end of the bound RNA substrate. The side chain of Leu18 is located between the bases of nucleotides U1 and U2 of the bound substrate. The phenyl ring of Tyr129 forms a hydrophobic interaction with the base of nucleotide U1. All these interacting residues act as a wedge that may allow *Cps*ORN to cleave the exact position of RNA substrate and guide the 3′-terminal exo-RNA cutting. These three residues interacting with the bound RNA bases are strictly conserved among other ORN homologs. These structural characteristics explain why *Cps*ORN has exo-RNase activity only for substrates that are 2–5 nucleotide long. It appears that the amino acid residues involved in substrate-binding interact primarily via hydrophobic interactions. Although the side chain of Cys62 interacts with the hydroxyl group of the ribose sugar, this cysteine is not conserved in other ORNs. Moreover, this unspecific interaction is relatively weak, with a distance of 3.5 Å. Based on these results, we conclude that *Cps*ORN does not distinguish between single-strand short DNA and short RNA in binding (Fig. 1A). However, the physiological meaning of the interaction between *Cps*ORN and single-strand short DNA is still unclear.

Furthermore, the structure of *Cps*ORN bound to two separated uridines was determined to understand how the cleaved products interact with *Cps*ORN (Fig. 5B). In this structure, two uridine mononucleotides are located in similar positions as the last two 3′-terminal nucleotides of the U-U complex structure. Both uridine molecules interact with the manganese ion. The O atom from the phosphate of U2 directly interacts with manganese, and the C3 atom of U1 is located near the manganese ion and the O atom of U2. This region matched the cleavage region of the oligoribonucleotide. This suggests that the last two 3′-terminal nucleotides (U1-U2) contain the cleavage phosphate site.

### RNA hydrolysis mechanism of *Cps*ORN

**Figure 6:**
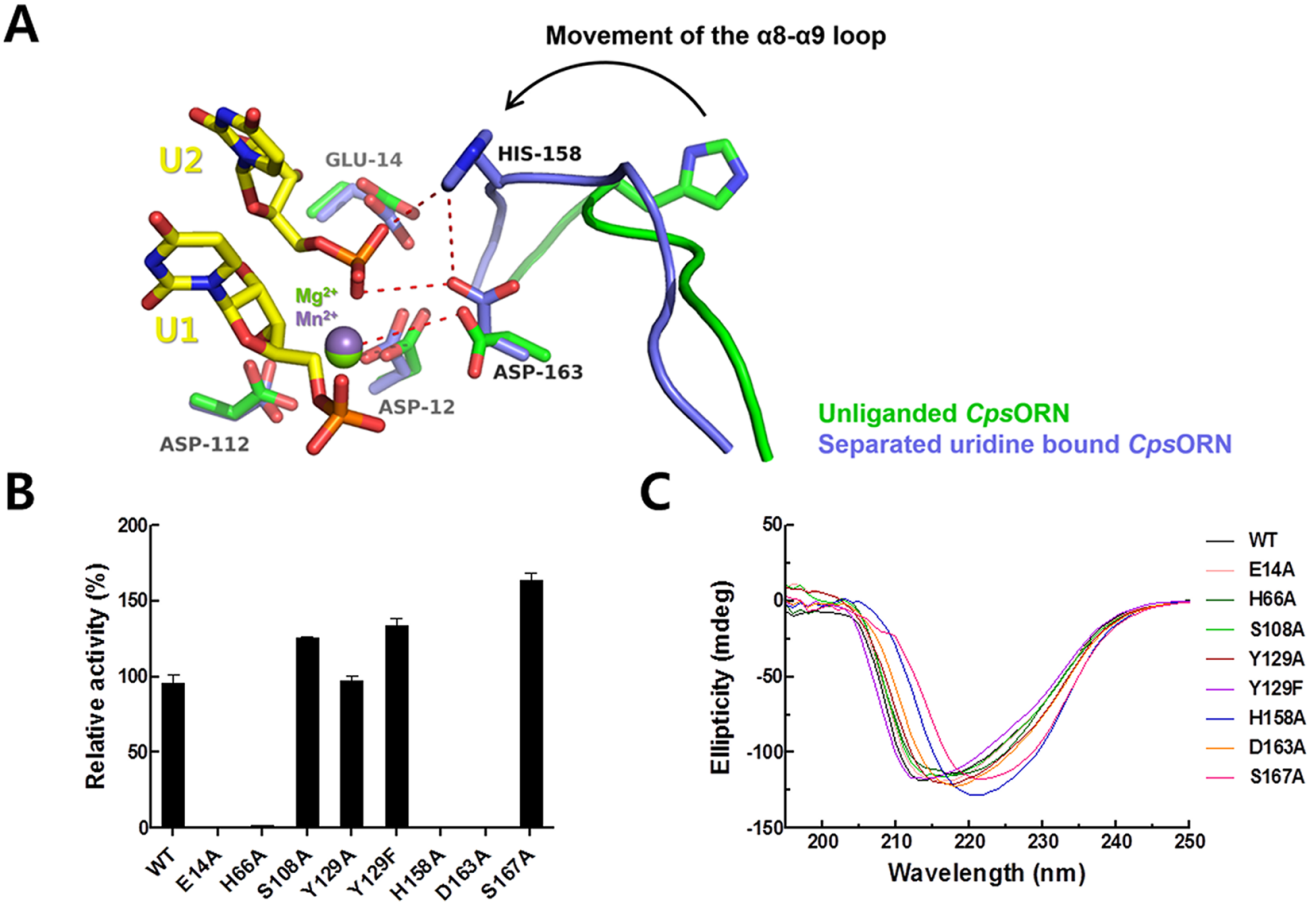
Small RNA hydrolysis mechanism of *Cps*ORN. (**A**) Structural comparison of unliganded *Cps*ORN and separated uridine-bound *Cps*ORN shows that the α8-α9 loop region undergoes movement upon ligand binding. (**B**) The relative enzyme activities of the mutants are calculated by taking the activity of wild-type *Cps*ORN as 100%. Experiments were performed in triplicate, and the error bar indicates the standard deviation. (**C**) CD spectra were recorded for wild-type and mutants in the range of 190–250 nm at 20 °C.

In both RNA-bound complex structures, an interesting movement of the His158 residue located on the α8-α9 loop region was observed (Fig. 6A). Structural comparison between separated uridine-bound structure and unliganded-structure clearly shows a prominent difference in their flexible loop region. The His158 residue showed inward movement toward the active site to interact with the cleaved uridine phosphate, in the two-uridine complex structure. It should be noted that the side chain of His158 is also near the cleavage site of the U-U RNA substrate. Thus, the His158 was suspected as an activator of the water molecule during the enzymatic reaction of *Cps*ORN. This speculation was further confirmed through a biochemical assay together with the site-directed mutation. In separated two-uridine-bound structure, manganese ion has a different interaction network compared with those of the U-U complex structure. In particular, the manganese ion forms a new interaction with the 3′-oxygen of the ribose in the U1 ribonucleotide. To identify the important residues involved in the enzymatic reaction, several mutant proteins were analysed by the *p*NP-TMP hydrolysis assay (Fig. 6B,C). As expected, the H158A mutant completely lost its cleavage ability. Interestingly, H66A mutant, located on the α2 helix, also lost its activity. This residue interacts with the carboxyl group of Glu14 residue and 3′-oxygen of the ribose of the U2 ribonucleotide. It was initially thought that His66 located near the active site, potentially stabilises the negative charge of the nucleotide. These inspections suggest that the His66 residue also plays a significant role in the cleavage mechanism. His66 and His158 are completely conserved among other ORNs. Two other candidate residues expected to be involved in the enzyme reaction were Ser108, Tyr129, and Ser167. However, mutants in which these two residues were targeted (S108A, Y129A, Y129F, and S167A) still maintained a similar enzyme activity as the wild-type *Cps*ORN. It suggests that the Ser108 and Tyr129 residues are not essential for the RNase activity of *Cps*ORN.

This study is the first report on the structure of substrate RNA-bound (two linked uridine) *Cps*ORN. We also report the structure of two separate uridine-bound forms that mimic the product bound state of *Cps*ORN. With the help of these structures, essential interactions, and conformational changes in the enzymatic reaction of *Cps*ORN could be identified. Furthermore, our *in vitro* RNase assay allowed us to confirm the important residues in substrate-binding and enzyme reaction of *Cps*ORN, supporting our structural findings. We view His158 as a likely candidate for activation of water molecule during the enzymatic reaction. The divalent metal ion might also be important during the catalytic reaction of *Cps*ORN. Probably, the metal ion can interact with negatively charged leaving group, thus, stabilising the transition state or assisting the RNA hydrolysis process by interaction with the attacking water molecule. This suggested mechanism is analogous to the previously proposed mechanism of RNase T functioning^41^. Our DALI search results show that *Cps*ORN has a structural similarity with other exonuclease structures (Supplementary Table S2). Interestingly, the binding mode of the last two RNA substrates is very similar among the DEDD exonuclease structures, suggesting that the RNA cleavage mechanism is well conserved within the DEDD exonuclease family. However, compared to *Cps*ORN, other exonuclease structures contain the longer and larger interaction surfaces for longer RNA-binding (Supplementary Fig. S1). Therefore, a relatively reduced RNA-binding area could explain why *Cps*ORN can accommodate and hydrolyse only small RNA. In conclusion, our structural analyses, together with the *in vitro* exoribonuclease activity assay, provide important and novel insights into the substrate-binding and cleaving mechanism of this unique exoribonuclease. It should be noted that the unliganded and two separated uridine-bound *Cps*ORN structures contain one metal ion and these structures do not have enough space for second metal binding (Supplementary Fig. S3). However, our present data cannot completely exclude the possibility of another metal binding in *Cps*ORN in the case of two metal-dependent exonucleases. Thus, understanding the precise enzyme reaction mechanism of *Cps*ORN requires higher resolution structural data and biochemical assays to confirm metal binding.

## Material and Methods

### Cloning and mutagenesis of *Cps*ORN

The full-length gene *orn* (GenBank AAZ28688.1) from the genomic DNA of *C. psychrerythraea* strain 34H was amplified by polymerase chain reaction (PCR), using the forward primer 5′-CGATAACATATGATGGCGGGTAACGAC-3′ and reverse primer 5′-CGATAAGAATTCCTATAACTTGAAGAAAAG -3′. The amplified DNA fragments were cloned into the pET-28a expression vector (Novagen, Madison, USA), using NdeI and XhoI restriction enzymes. The resulting construct contains N-terminal hexahistidine tag and a thrombin protease recognition site. After confirming the sequence, the vectors were transformed into *E. coli* strain DH5α for storage and into strain BL21(DE3) for protein expression. The catalytic residue of Asp163 was replaced by alanine. Site-directed mutagenesis was performed by PCR using the following mutagenic primers (5′-CACTTAGCCCTAGATGCCATCAAAGAGTCGATCG-3′ and 5′-CGATCGACTCTTTGAT GGCATCTAGGGCTAAGTG-3′). Wild-type containing plasmid was used as the template in PCR. Upon confirmation of the plasmid sequence, it was transfected into *E. coli* strain DH5α for storage and into strain BL21(DE3) for protein expression.

### Expression and purification of *Cps*ORN and mutants

The *Cps*ORN and the mutants were expressed and purified using the same procedure. The cells were grown in 4 L of LB medium supplemented with kanamycin (50 µg mL^−1^) at 37 °C. Once the OD_600_ approached 0.7, overnight expression was induced with 0.5 mM isopropyl-1-thio-β-D-galactopyranoside (IPTG) at 25 °C. The cells were collected by centrifugation at 6000 rpm and 4 °C for 20 m and resuspended in lysis buffer (50 mM sodium phosphate, 300 mM NaCl, 5 mM imidazole pH 8.0) with 0.2 mg mL^−1^ lysozyme. After sonication on ice and centrifugation at 16000 rpm for 1 h at 4 °C, the cleared supernatant was poured into a pre-equilibrated Ni-NTA column (Qiagen, Hilden, Germany). The unbound protein was flowed through and the poly-histidine-tag-fused proteins bound to Ni-NTA resin were washed with ten bed-column volume of wash buffer (50 mM sodium phosphate, 300 mM NaCl, 20 mM imidazole pH 8.0). The proteins were then eluted with elution buffer (50 mM sodium phosphate, 300 mM NaCl, 300 mM imidazole pH 8.0). The proteins were concentrated using an Amicon Ultra Centrifugal Filter (Ultracel-10K; Millipore, Darmstadt, Germany). The poly-histidine tag was cleaved by overnight incubation at 4 °C with thrombin and then loaded onto a Superdex 200 column (GE Healthcare, Piscataway, USA) pre-equilibrated with 20 mM Tris-HCl pH 8.0, 150 mM NaCl. The fractions containing wild-type *Cps*ORN were collected and concentrated to 20.7 mg mL^−1^, using Amicon Ultra Centrifugal Filters.

Mutations were introduced by site-directed PCR mutagenesis using mutagenic primers (Supplemental Table S1). Plasmids for wild-type *Cps*ORN were used as the template DNA for mutagenesis. After amplification, the PCR fragments were digested by the DpnI restriction enzyme to eliminate template plasmids. The nucleotide sequences of the desired mutants were confirmed by DNA sequencing. All mutant *Cps*ORN proteins were expressed and purified using the same procedure used for the wild-type *Cps*ORN.

### Gel-based enzyme activity assay

*Cps*ORN activities on 5-mer RNA or DNA were determined using 100 pmol custom-made 5′-fluorescein-labelled oligo in a buffer consisting of 50 mM Tris-HCl (pH 8.0) and 5 mM MnCl_2_. After 5 m pre-incubation, the reaction was initiated by the addition of 20 ng protein at 25 °C. At different time points, aliquots were taken and stopped with an equal volume of loading buffer (4 x Tris-borate-EDTA, 100 mM DTT, 16% glycerol, 20 mM EDTA, and 0.04% bromophenol blue) on ice. Each sample was resolved on 28% polyacrylamide gel at 5 V/cm and the gel was visualised using Gel Doc™ EZ System (Bio-Rad, USA). Assay on 24-mer RNA oligo was also performed in the same way as described above, except that the samples were loaded onto 20% PAA gel.

### Hydrolysis of *p*NP-TMP

Hydrolysis activities of *Cps*ORN wild-type and mutants on *p*NP-TMP were determined by spectrophotometrically measuring the production rate of the *p*-nitrophenolate anion, as described previously^42^. Briefly, standard reactions were performed in 50 mM Tris buffer (pH 8.0) containing 150 mM NaCl and 3 mM *p*NP-TMP at 25 °C, controlled by a water-bath thermostat. After equilibration for 5 min, 1 mM MnCl_2_ and 400 nM protein were added to start the reaction. Absorbance was measured at 420 nm by UV spectrophotometer (UV1800, Shimadzu, Japan).

### Crystallisation and data collection

Initial crystallisation screenings for unliganded form (wild-type), *p*NP-TMP-bound form (D163A mutant), two separated uridine-bound form (wild-type), and five linked uridine (D163A mutant)-bound form were performed using a mosquito crystallisation robot (TTP Labtech, UK). Commercially available crystallisation solution kits, such as MCSG I-IV (Microlytic, Burlington, USA), Wizard Classic I-IV (Emerald Bio, Seattle, USA), Index, SaltRx, PEG/Ion (Hampton Research, USA), and SG-1 (Molecular Dimensions, USA) were used. For preparing each liganded form, D163A mutant protein was mixed with 5 mM *p*NP-TMP or 5 mM RNA (5′-UUUUU-3′) substrate, and 5 mM uridine was added to the wild-type protein solution. Except for the unliganded form, all samples were supplemented with 1 mM manganese chloride. Sitting-drop vapour-diffusion method was carried out at 293 K in a 96-well crystallisation plate (Emerald Bio, Bainbridge Island, WA). A 200-nL volume of the protein solution was mixed with 200 nL reservoir solution and equilibrated against 80 µL reservoir solution. The crystals of unliganded *Cps*ORN were obtained from 15% (w/v) PEG 3350 and 0.1 M magnesium formate (MCSG I #G10). Furthermore, to make larger single crystals, the drop volume was increased from 200 nL to 1 μL against 500 μL reservoir solution at 293 K, using the hanging-drop vapour-diffusion method in 24-well plates. As a result, optimised single crystals were obtained in the same condition as that of initial screening. The crystals of *p*NP-TMP-bound *Cps*ORN were observed under several conditions. Among them, the most suitable crystals were obtained from the 1.4 M ammonium tartrate dibasic and 0.1 M Tris pH 8.5 (SaltRx #G12) condition. The crystals of linked RNA-bound form were obtained with 0.04 M citric acid, 0.06 M Bis-Tris propane pH 6.4, and 20% (w/v) PEG 3350 (PEG/Ion #H3). Crystals of uridine-bound form were obtained with 0.2 M potassium thiocyanate and 20% (w/v) PEG 3350 (SG-1 #D4). All single crystals were harvested with a cryoloop and protected from the liquid-nitrogen gas stream using the Paratone-N oil (Hampton Research, Aliso Viejo, USA). Complete datasets of unliganded form, *p*NP-TMP-bound form, linked uridine-bound form, and separated uridine-bound form were collected on BL-5C beamline of the Pohang Accelerator Laboratory (PAL; Pohang, Korea). Datasets of unliganded form and linked uridine-bound form contained 360 images, whereas that of *p*NP-TMP-bound form and separated uridine-bound form contained 200 and 120 images, respectively. Datasets for all forms were collected with an oscillation range of 1° per image with 1 s exposure. The data were processed and scaled using the HKL-2000 program^43^. The data collection statistics are summarised in Table 1.

**Table 1:** X-ray diffraction data collection and refinement statistics.

Data set	*Cps*ORN (wild-type)	*p*NP-TMP bound *Cps*ORN (D163A mutant)	Linked uridine-bound *Cps*ORN (D163A mutant)	Separated uridine-bound *Cps*ORN (wild-type)
X-ray source	PAL 5C beamline	PAL 5C beamline	PAL 5C beamline	PAL 5C beamline
Space group	*P*4_1_22	*P*3_2_21	*P*3_2_21	*P*3_2_21
Unit cell parameters (Å, °)	a = b = 53.112, c = 143.338, α = β = γ = 90	a = b = 59.532, c = 236.269, α = β = 90, γ = 120	a = b = 59.667, c = 236.201, α = β = 90, γ = 120	a = b = 59.285, c = 234.16, α = β = 90, γ = 120
Wavelength (Å)	0.9795	0.9795	0.9795	0.9796
Resolution (Å)	50.00–2.70 (2.75–2.70)	50.00–2.20 (2.24–2.20)	50.00–2.45 (2.49–2.45)	50.00–2.21 (2.25–2.21)
Total reflections	139608	246445	350650	103571
Unique reflections	6155 (281)	25387 (1231)	18200 (948)	21675 (1043)
Average I/σ (I)	56.6 (5.2)	38.5 (5.5)	76.1 (11.0)	43.9 (5.0)
*R*_merge_^a^	0.096 (0.509)	0.117 (0.669)	0.089 (0.451)	0.065 (0.438)
Redundancy	22.7 (26.0)	9.7 (10.9)	21.8 (19.3)	4.8 (4.8)
Completeness (%)	99.8 (100.0)	97.8 (100.0)	96.2 (100.0)	86.2 (86.4)
**Refinement**				
Resolution range (Å)	35.52–2.70 (3.40–2.70)	47.25–2.19 (2.25–2.19)	47.34–2.45 (2.58–2.45)	47.02–2.21 (2.26–2.21)
No. of reflections of working set	6088 (2818)	24032 (1699)	18109 (2536)	20644 (1473)
No. of reflections of test set	303 (131)	1296 (86)	927 (115)	1031 (77)
No. of amino acid residues	180	366	362	366
No. of water molecules	23	147	60	181
*R*_cryst_^b^	0.255 (0.299)	0.204 (0.260)	0.237 (0.274)	0.193 (0.276)
*R*_free_^c^	0.297 (0.394)	0.271 (0.323)	0.276 (0.346)	0.270 (0.413)
R.m.s. bond length (Å)	0.010	0.020	0.008	0.016
R.m.s. bond angle (°)	1.129	1.965	1.204	1.884
Average B value (Å^2^) (protein)	78.60	53.55	62.90	58.23
Average B value (Å^2^) (solvent)	75.70	53.87	58.65	57.41

^a^R_merge_ = ∑|<I> − I|/∑<I>.

^b^R_cryst_ = ∑||Fo| − |Fc||/∑|Fo|.

^c^R_free_ calculated with 5% of all reflections excluded during the refinement stages using high-resolution data.

Values in parentheses refer to the highest resolution shells.

### Structure determination and refinement of *Cps*ORN

The crystal structure of unliganded *Cps*ORN (wild-type) was solved by molecular replacement using *MOLREP*^44^. Oligoribonuclease from *H. influenzae* (PDB code 1J9A) with a 55% sequence identity was used as a template. Matthews coefficient calculations predicted that one molecule is present in the asymmetric unit, with a Matthew coefficient of 2.45 Å^3^ Da^−1^ and 49.79% solvent content^45^. The model was then iteratively refined and rebuilt using COOT and *REFMAC5* from the *CCP4* suite, and *phenix.refine* from *PHENIX* suite^46–49^. The final model had a *R*_cryst_ of 23.51% and a *R*_free_ of 29.69%, with a total of 180 amino acid residues and 30 water molecules. All other forms of *Cps*ORN were solved by molecular replacement, using the unliganded *Cps*ORN as the template. Successive refinement and model rebuilding were then performed along the same lines as unliganded *Cps*ORN. The *MolProbity* structure validation server was used to check the qualities of all models^50^. The final models and reflection data were deposited in the Protein Data Bank under the accession codes of 6A4A (unliganded *Cps*ORN), 6A4D (*p*NP-TMP-bound *Cps*ORN), 6A4E (linked uridine-bound *Cps*ORN), and 6A4F (separated uridine-bound *Cps*ORN), respectively. The detailed refinement statistics are listed in Table 1. *PyMOL* was used for the visualisation and production of the figures^51^.

### Circular-dichroism (CD) spectroscopy

The CD spectra were recorded from 250 to 195 nm (0.1 nm intervals, 1 nm bandwidth) using Chirascan Circular-Dichroism Spectropolarimeter (Applied Photophysics, Surrey, UK). Protein solution (1 mg/mL) in 20 mM Tris-HCl (pH 8.0) and 150 mM NaCl were loaded onto 0.1 cm path length cuvette. The spectra were collected in triplicate and normalised by subtraction of the background scan with buffer. During thermal unfolding, the CD melting curves were obtained by plotting changes in ellipticity at 222 nm over a temperature range of 5–95 °C at intervals of 1 °C. The melting temperature (T_m_) was defined as the point at which 50% of the sample denatured.

## Supplementary information

Supplementary tables and figures

## References

[1] Houseley J, Tollervey D (2009). The many pathways of RNA degradation. Cell.

[2] Kushner SR (2002). mRNA decay in Escherichia coli comes of age. Journal of bacteriology.

[3] Jain C (2002). Degradation of mRNA in Escherichia coli. IUBMB life.

[4] Condon C (2003). RNA processing and degradation in Bacillus subtilis. Microbiology and Molecular Biology Reviews.

[5] Deutscher MP (2006). Degradation of RNA in bacteria: comparison of mRNA and stable RNA. Nucleic acids research.

[6] Kaplan R, Apirion D (1975). Decay of ribosomal ribonucleic acid in Escherichia coli cells starved for various nutrients. Journal of Biological Chemistry.

[7] Bessarab DA, Kaberdin VR, Wei C-L, Liou G-G, Lin-Chao S (1998). RNA components of Escherichia coli degradosome: evidence for rRNA decay. Proceedings of the National Academy of Sciences.

[8] Bernstein JA, Lin P-H, Cohen SN, Lin-Chao S (2004). Global analysis of Escherichia coli RNA degradosome function using DNA microarrays. Proceedings of the National Academy of Sciences of the United States of America.

[9] Li, Z. & Deutscher, M. P. Exoribonucleases and Endoribonucleases. *EcoSal Plus* **1** (2004). 10.1128/ecosalplus.4.6.3 26443351

[10] Donovan WP, Kushner SR (1986). Polynucleotide phosphorylase and ribonuclease II are required for cell viability and mRNA turnover in Escherichia coli K-12. Proceedings of the National Academy of Sciences.

[11] Cheng Z-F, Zuo Y, Li Z, Rudd KE, Deutscher MP (1998). The vacB Gene Required for Virulence inShigella flexneri and Escherichia coli Encodes the Exoribonuclease RNase R. Journal of Biological Chemistry.

[12] Niyogi S, Datta A (1975). A novel oligoribonuclease of Escherichia coli. I. Isolation and properties. Journal of Biological Chemistry.

[13] Ghosh S, Deutscher MP (1999). Oligoribonuclease is an essential component of the mRNA decay pathway. Proceedings of the National Academy of Sciences.

[14] Zhang X, Zhu L, Deutscher MP (1998). Oligoribonuclease is encoded by a highly conserved gene in the 3 3gella flexneri and Escherich. Journal of bacteriology.

[15] Orr MW (2015). Oligoribonuclease is the primary degradative enzyme for pGpG in Pseudomonas aeruginosa that is required for cyclic-di-GMP turnover. Proceedings of the National Academy of Sciences.

[16] Cohen D (2015). Oligoribonuclease is a central feature of cyclic diguanylate signaling in Pseudomonas aeruginosa. Proceedings of the National Academy of Sciences.

[17] Chen G (2016). Oligoribonuclease is required for the type III secretion system and pathogenesis of Pseudomonas aeruginosa. Microbiological research.

[18] Zuo Y, Deutscher MP (2001). Exoribonuclease superfamilies: structural analysis and phylogenetic distribution. Nucleic acids research.

[19] Moser MJ, Holley WR, Chatterjee A, Mian IS (1997). The proofreading domain of Escherichia coli DNA polymerase I and other DNA and/or RNA exonuclease domains. Nucleic acids research.

[20] Steitz TA, Steitz JA (1993). A general two-metal-ion mechanism for catalytic RNA. Proceedings of the National Academy of Sciences.

[21] Yang W, Lee JY, Nowotny M (2006). Making and breaking nucleic acids: two-Mg2+-ion catalysis and substrate specificity. Molecular cell.

[22] Bernad A, Blanco L, Lázaro J, Martin G, Salas M (1989). A conserved breaking nucleic acids: two-Mg2+-ion catalysis and subst. Cell.

[23] Barnes MH, Spacciapoli P, Li DH, Brown NC (1995). The 3 3cids: two-Mgse site of DNA polymerase III from gram-positive bacteria: definition of a novel motif structure. Gene.

[24] Koonin EV (1997). A conserved ancient domain joins the growing superfamily of 3 3e site of DNA pol*Current*. Biology.

[25] Nguyen LH, Erzberger JP, Root J, Wilson DM (2000). The human homolog of Escherichia coli Orn degrades small single-stranded RNA and DNA oligomers. Journal of Biological Chemistry.

[26] Franklin MC (2015). Structural genomics for drug design against the pathogen Coxiella burnetii. Proteins: Structure, Function, and Bioinformatics.

[27] Chin KH, Yang CY, Chou CC, Wang AHJ, Chou SH (2006). The crystal structure of XC847 from Xanthomonas campestris: a 3′–5′ oligoribonuclease of DnaQ fold family with a novel opposingly shifted helix. Proteins: Structure, Function, and Bioinformatics.

[28] Methé BA (2005). The psychrophilic lifestyle as revealed by the genome sequence of Colwellia psychrerythraea 34H through genomic and proteomic analyses. Proceedings of the National Academy of Sciences of the United States of America.

[29] Huston, A. L., Krieger-Brockett, B. B. & Deming, J. W. J. E. M. Remarkably low temperature optima for extracellular enzyme activity from Arctic bacteria and sea ice. **2**, 383–388 (2000). 10.1046/j.1462-2920.2000.00118.x 11234926

[30] Methé, B. A. *et al*. The psychrophilic lifestyle as revealed by the genome sequence of Colwellia psychrerythraea 34H through genomic and proteomic analyses. **102**, 10913–10918 (2005). 10.1073/pnas.0504766102 PMC1180510 16043709

[31] Do, H. *et al*. Crystal structure of UbiX, an aromatic acid decarboxylase from the psychrophilic bacterium Colwellia psychrerythraea that undergoes FMN-induced conformational changes. **5**, 8196 (2015). 10.1038/srep08196 PMC4316190 25645665

[32] Do, H. *et al*. Crystal structure and comparative sequence analysis of GmhA from Colwellia psychrerythraea strain 34H provides insight into functional similarity with DiaA. **38**, 1086 (2015). 10.14348/molcells.2015.0191 PMC4697000 26612680

[33] Lee JH, Choi JM, Kim HJ (2017). J. B. & communications, b. r. Crystal structure of 5-enolpyruvylshikimate-3-phosphate synthase from a psychrophilic bacterium. Colwellia psychrerythraea 34H..

[34] Park, S.-H. *et al*. Crystal structure and functional characterization of an isoaspartyl dipeptidase (CpsIadA) from Colwellia psychrerythraea Sin 34H. **12**, e0181705 (2017). 10.1371/journal.pone.0181705 PMC5517026 28723955

[35] Datta A, Niyogi K (1975). A novel oligoribonuclease of Escherichia coli. II. Mechanism of action. Journal of Biological Chemistry.

[36] Yu D, Deutscher MP (1995). Oligoribonuclease is distinct from the other known exoribonucleases of Escherichia coli. Journal of bacteriology.

[37] Mechold U, Ogryzko V, Ngo S, Danchin A (2006). Oligoribonuclease is a common downstream target of lithium-induced pAp accumulation in Escherichia coli and human cells. Nucleic acids research.

[38] Park AY (2008). Hydrolysis of the 5ch V., Ngo, S. & Danchin, A. Oligoribonuclease is a common downstream target of lithium-induced pAp accumulation in. Protein expression and purification.

[39] Santiago M, Ramírez-Sarmiento CA, Zamora RA, Parra LP (2016). Discovery, molecular mechanisms, and industrial applications of cold-active enzymes. Frontiers in microbiology.

[40] Lonhienne T, Gerday C, Feller G (2000). Psychrophilic enzymes: revisiting the thermodynamic parameters of activation may explain local flexibility. Biochimica et Biophysica Acta (BBA)-Protein Structure and Molecular Enzymology.

[41] Hsiao Y-Y, Duh Y, Chen Y-P, Wang Y-T, Yuan HS (2012). How an exonuclease decides where to stop in trimming of nucleic acids: crystal structures of RNase T–product complexes. Nucleic acids research.

[42] Hamdan S (2002). Hydrolysis of the 5′-p-Nitrophenyl Ester of TMP by the Proofreading Exonuclease (ε) Subunit of Escherichia coli DNA Polymerase III. Biochemistry.

[43] Otwinowski, Z. & Minor, W. In *Methods in enzymology* Vol. 276, 307–326 (Elsevier, 1997). 10.1016/S0076-6879(97)76066-X 27754618

[44] Vagin A, Teplyakov A (1997). MOLREP: an automated program for molecular replacement. Journal of applied crystallography.

[45] Kantardjieff KA, Rupp B (2003). Matthews coefficient probabilities: improved estimates for unit cell contents of proteins, DNA, and protein–nucleic acid complex crystals. Protein Science.

[46] Emsley P, Cowtan K (2004). Coot: model-building tools for molecular graphics. Acta Crystallographica Section D: Biological Crystallography.

[47] Murshudov GN (2011). REFMAC5 for the refinement of macromolecular crystal structures. Acta Crystallographica Section D: Biological Crystallography.

[48] Winn MD (2011). Overview of the CCP4 suite and current developments. Acta Crystallographica Section D: Biological Crystallography.

[49] Afonine PV (2012). Towards automated crystallographic structure refinement with phenix. refine. Acta Crystallographica Section D: Biological Crystallography.

[50] Chen VB (2010). MolProbity: all-atom structure validation for macromolecular crystallography. Acta Crystallographica Section D: Biological Crystallography.

[51] DeLano, W. L. The PyMOL molecular graphics system. http://pymol.org (2002).

[52] Schuck P (2000). Size-distribution analysis of macromolecules by sedimentation velocity ultracentrifugation and lamm equation modeling. Biophysical journal.

[53] Schuck P, Rossmanith P (2000). Determination of the sedimentation coefficient distribution by least-squares boundary modeling. Biopolymers.

