# Supplementary material for: Structural basis of small RNA hydrolysis by oligoribonuclease (CpsORN) from Colwellia psychrerythraea strain 34H
Source: Sci Rep. 2019 Feb 25;9:2649. doi: 10.1038/s41598-019-39641-0 (PMC6390093; doi:10.1038/s41598-019-39641-0)
Supplement: Supplementary file 1 — Supplementary tables and figures [file 41598_2019_39641_MOESM1_ESM.pdf]

Supplementary Information for

Structural basis of small RNA hydrolysis by oligoribonuclease (*Cps*ORN) from *Colwellia psychrerythraea* strain 34H

Chang Woo Lee[1,2#], Sun-Ha Park[1#], Chang-Sook Jeong[1,2], Sun-Shin Cha[3], Hyun Park[1,2], Jun Hyuck Lee[1,2]*

[1]Unit of Polar Genomics, Korea Polar Research Institute, Incheon, Republic of Korea; [2]Department of Polar Sciences, University of Science and Technology, Incheon, Republic of Korea; [3]Department of Chemistry & Nano Science, Ewha Woman's University, Seoul, Republic of Korea

[#]CWL and SHP contributed equally to this work.

*To whom correspondence should be addressed: Dr Jun Hyuck Lee: Unit of Polar Genomics, Korea Polar Research Institute, 26, Songdomiare-ro, Yeonsu-gu, Incheon, 21990, Republic of Korea; junhyucklee@kopri.re.kr; Tel. (+82) 32 760 5555; FAX. (+82) 32 760 5509.

Supplementary Table S1. Primers used in this study.

Primer	Direction	Sequence (5′→3′)
*Cps*ORN_WT	Forward	CGATAACATATGATGGCGGGTAACGAC
*Cps*ORN_WT	Reverse	CGATAAGAATTCCTATAACTTGAAGAA AAG
*Cps*ORN_E14A mutant	Forward	GGCTTGATTTGGCAATGACAGGCCTC
*Cps*ORN_E14A mutant	Reverse	GAGGCCTGTCATTGCCAAATCAAGCC
*Cps*ORN_H66A mutant	Forward	CATGGTGCATTGAACATGCCGGGAAATCAGGATTAAC
*Cps*ORN_H66A mutant	Reverse	GTTAATCCTGATTTCCCGGCATGTTCAATGCACCATG
*Cps*ORN_S108A mutant	Forward	CACCCATGTGTGGTAATGCCATCGGTCAAGATAGAC
*Cps*ORN_S108A mutant	Reverse	GTCTATCTTGACCGATGGCATTACCACACATGGGTG
*Cps*ORN_Y129A mutant	Forward	GATTTTGAAGATCATTTCCATGCCCGAAACTTAGATGT TAGTAC
*Cps*ORN_Y129A mutant	Reverse	GTACTAACATCTAAGTTTCGGGCATGGAAATGATCTTC AAAATC
*Cps*ORN_Y129F mutant	Forward	GATTTTGAAGATCATTTCCATTTCCGAAACTTAGATGT TAGTAC
*Cps*ORN_Y129F mutant	Reverse	GTACTAACATCTAAGTTTCGGAAATGGAAATGATCTTC AAAATC
*Cps*ORN_H158A mutant	Forward	GTTAAAACGGGGGCTGCCTTAGCCCTAGATGAC
*Cps*ORN_H158A mutant	Reverse	GTCATCTAGGGCTAAGGCAGCCCCCGTTTTAAC
*Cps*ORN_D163A mutant	Forward	CACTTAGCCCTAGATGCCATCAAAGAGTCGATCG
*Cps*ORN_D163A mutant	Reverse	CGATCGACTCTTTGATGGCATCTAGGGCTAAGTG
*Cps*ORN_S167A mutant	Forward	CTAGATGACATCAAAGAGGCGATCGCTGAGTTAAAAG
*Cps*ORN_S167A mutant	Reverse	CTTTTAACTCAGCGATCGCCTCTTTGATGTCATCTAG

Supplementary Table S2. Structural homolog search result for *Cps*ORN, from a DALI search (DALI-Lite server).

Protein	PDB code	DALI score	UniProtKB code	Sequence % ID with *Cps*ORN (aligned residue number)	Reference
Oligoribonuclease from *Escherichia coli*	1YTA	28.6	P0A784	55	Not yet published
Oligoribonuclease from *Haemophilus influenzae*	1J9A	28.3	P45340	55	Not yet published
Oligoribonuclease from *Acinetobacter baumannii*	5CY4	27.7	V5VGJ9	53	Not yet published
Oligoribonuclease from *Coxiella burnetii*	3TR8	27.6	Q83C93	50	(2015) Proteins 83: 2124-2136
Oligoribonuclease from *Xanthomonas campestris*	2GBZ	27.3	Q8P8S1	52	(2006) Proteins 65: 1036-1040
Oligoribonuclease from *Escherichia coli*	2IGI	27.1	P0A784	55	Not yet published
dT13 oligonucleotide bound exodeoxyribonulcease I from *Escherichia coli*	4JS5	16.2	P04995	21	(2013) Nucleic Acids Res. 41: 5887-5897
5cy-dT13 bound exodeoxyribonulcease I from *Escherichia coli*	4JRP	16.1	P04995	20	(2013) Nucleic Acids Res. 41: 5887-5897
ssDNA(AAA) bound RNase T from *Escherichia coli*	3V9X	14.0	P30014	19	(2012) Nucleic Acids Res. 40: 8144-8154
DNA polymerase III epsilon subunit from *Escherichia coli*	2IDO	14.0	P03007	15	(2006) J.Biol.Chem. 281: 38466-38471
RNase T from *Pseudomonas aeruginosa*	2F96	13.9	Q9HY82	19	(2007) Structure 15: 417-428

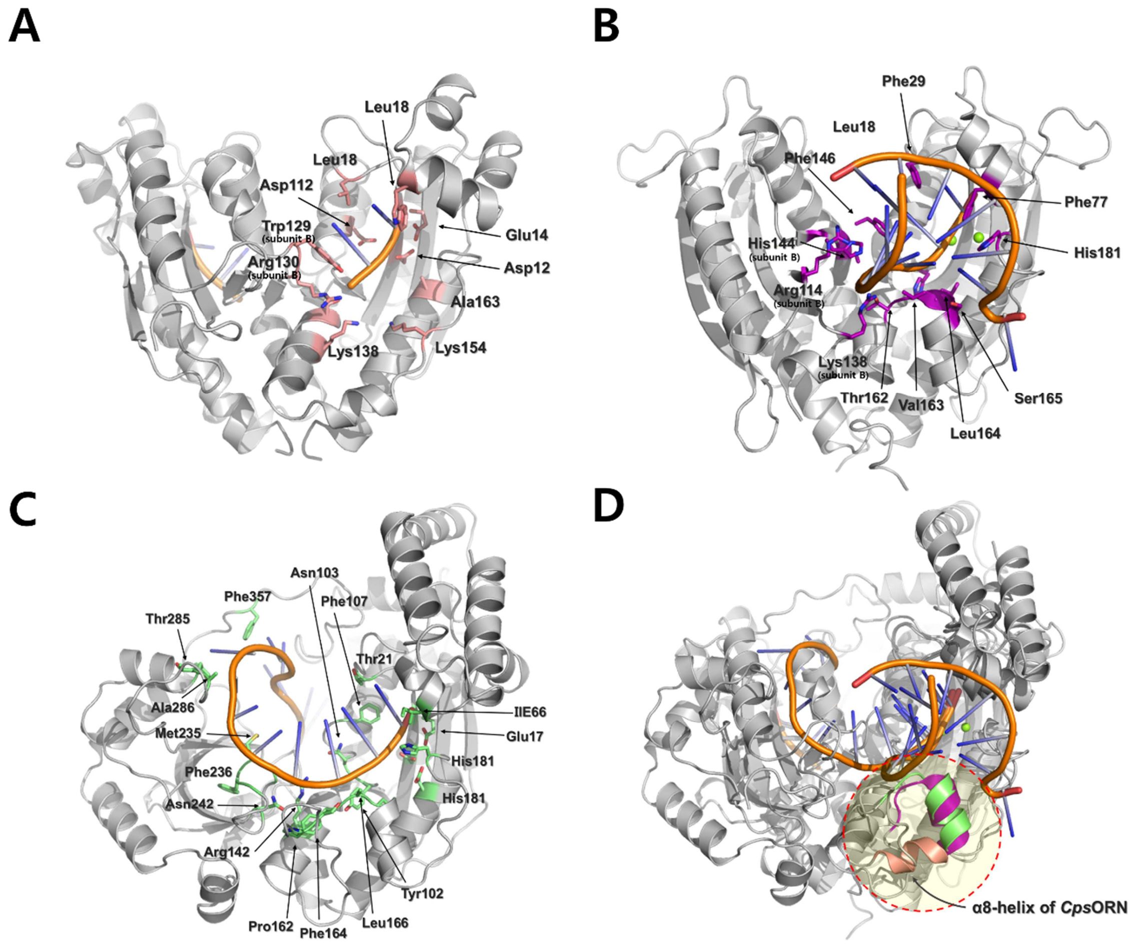

Supplementary Figure S1. Nucleotide binding interactions of several DEDD exonucleases. (A) Two linked uridine-binding mode of *Cps*ORN (D163A mutant). Interaction residues are shown as a salmon stick model. (B) A Y-structured DNA-bound complex of RNase T from *Escherichia coli* (PDB code 4KAZ). The residues that participate in the interaction with dsDNA are shown as a purple stick model. (C) A dA16 oligonucleotide-bound Exonuclease I from *Escherichia coli* (PDB code 4JS4). The interacting residues are presented in the form of a lime stick model. (D) Structural superposition of two linked uridine-binding mode of *Cps*ORN with DNA-bound complex of RNase T from *Escherichia coli* (PDB code 4KAZ) and a dA16 oligonucleotide-bound Exonuclease I from *Escherichia coli* (PDB code 4JS4). Comparison of the three models shows structural differences in the α-8 helix regions (red circle).

Supplementary Figure S2. Full-length gels of Figure 1A. Time course of oligoribonuclease activity of *Cps*ORN wild-type and D163A mutant on 5'-fluorescein-labelled RNA or DNA substrates.

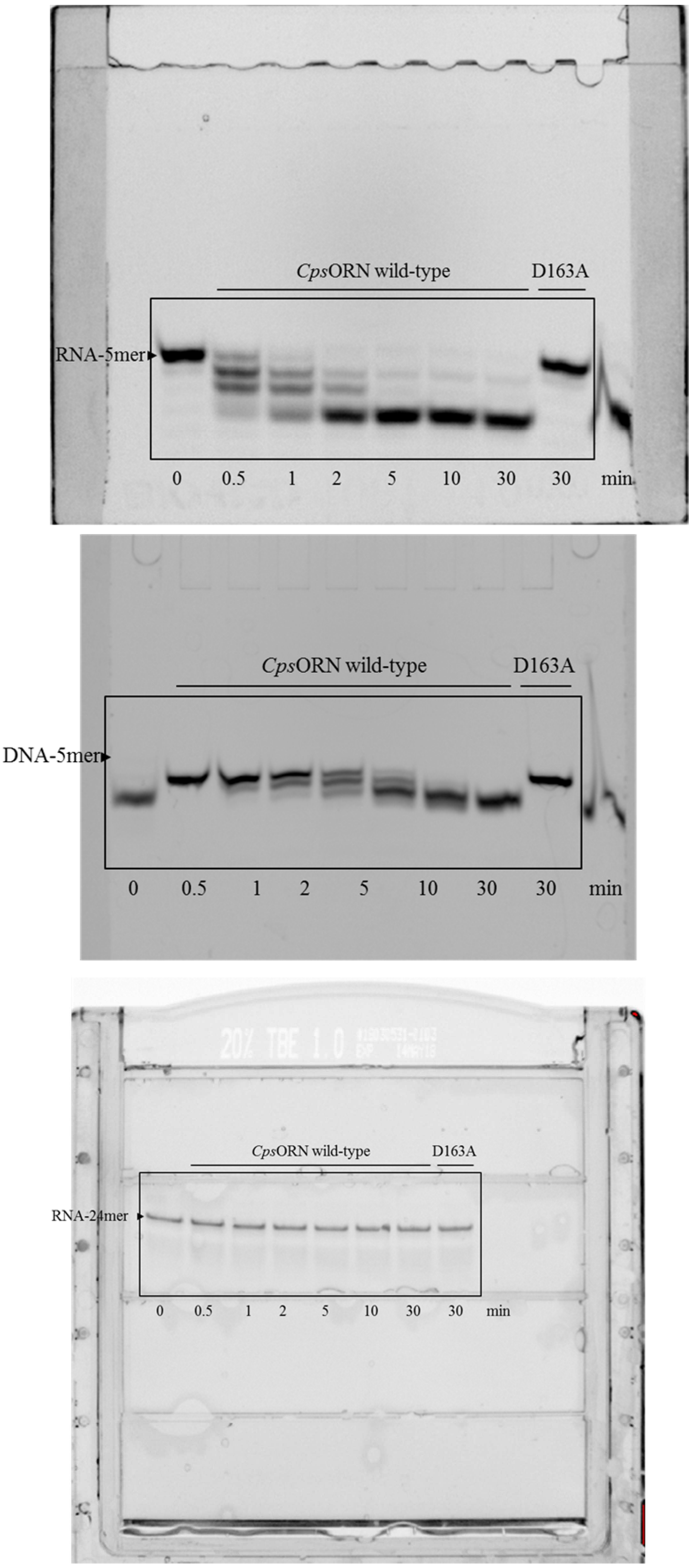

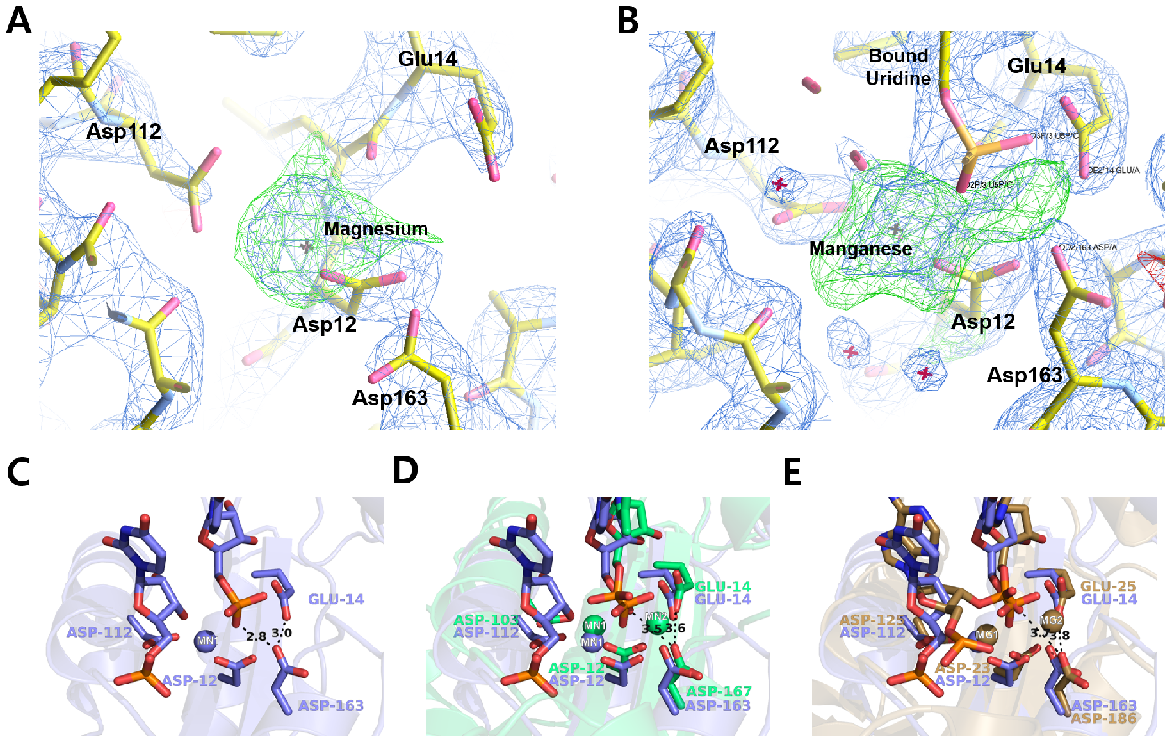

Supplementary Figure S3 Metal binding site of *Cps*ORN. (A) The omit map of unliganded *Cps*ORN structure clearly shows that one magnesium ion binds. (B) The omit map of separated uridine-bound *Cps*ORN structure also shows that only one distinct metal-binding site was observed. 2Fo-Fc density (blue) and Fo-Fc positive density (green) maps are contoured at 1.5 and 3.0 σ, respectively. (C) Metal binding active sites of *Cps*ORN (slate) are represented using a stick model. The distance between Glu14 and Asp163 is 3.0 Å, and the distance between phosphate of uridine and Asp163 is only 2.8 Å. This space is physically limited for metal binding. (D, E) Moreover, structural comparison analysis with two metal bound DNA polymerase III epsilon subunit (PDB code 2IDO; lime green) and ssDNA bound RNaseT (PDB code 3V9X; sand) show that these distances are relatively narrow.
